# Women and Infectious Diseases

**DOI:** 10.3201/eid1011.040800

**Published:** 2004-11

**Authors:** Julie L. Gerberding

**Affiliations:** * Centers for Disease Control and Prevention, Atlanta, Georgia, USA

**Keywords:** women, infectious disease, HIV/AIDS, malaria, commentary

Dr. Gerberding (Figure) is Director of the Centers for Disease Control and Prevention and the Administrator of the Agency for Toxic Substances and Disease Registry, Atlanta, Georgia, USA.

**Figure:**
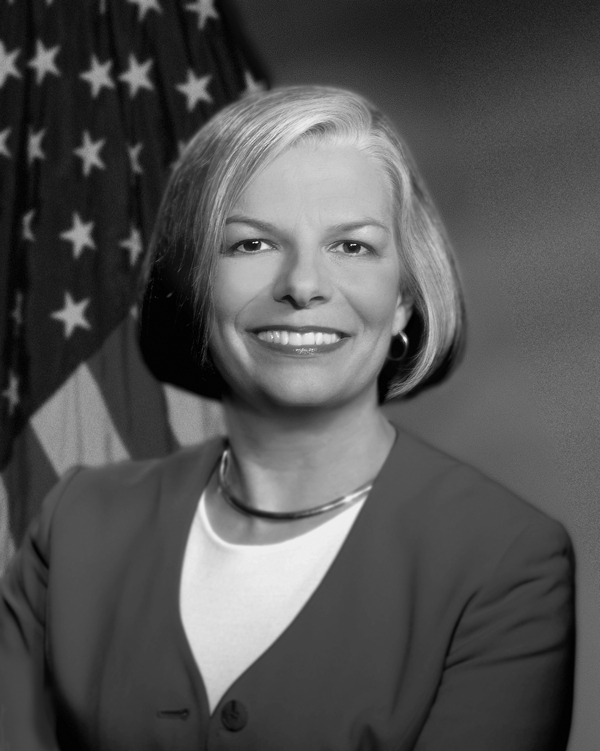
Dr. Gerberding is Director of the Centers for Disease Control and Prevention and the Administrator of the Agency for Toxic Substances and Disease Registry, Atlanta, Georgia, USA.

Social, economic, environmental, and demographic changes during the 20th century have affected the health of women. Many of the changes have benefited women's health, but some have had deleterious effects. Infectious diseases pose an especially formidable threat to women, claiming >15 million lives around the globe each year (*1*). For many infectious diseases, women are at higher risk and have more severe course of illness than men for many reasons, including biologic differences, social inequities, and restrictive cultural norms. These are often the same factors responsible for the disproportionate disease incidence among vulnerable populations throughout the world. Efforts to recognize and reduce health disparities among women have particular relevance for global health.

## HIV and AIDS in Women

In addition to hunger, lack of education, and environmental and sociocultural constraints, HIV/AIDS and malaria, along with tuberculosis, continue to disproportionately affect and further weaken the condition of women in many of the world's poorest regions. Recent estimates indicate that more than half of the estimated 38 million cases of adult HIV infection worldwide are in women (*2*). Moreover, the social, economic, and psychologic effects of the disease are more severe for women. When their partners or fathers die, women often lose economic rights. A Ugandan survey found that one in four widows reported losing their property after their partner died (*3*).

In sub-Saharan Africa, the region most affected by HIV, women are 30% more likely than men to be HIV-infected (*2*). The largest gender difference occurs among younger age groups. New HIV infections among women are also on the rise in the United States. An analysis of newly diagnosed HIV infections that occurred in 29 states between 1999 and 2002 showed that more than one third (35%) of cases resulted from heterosexual contact; among these heterosexually transmitted infections, almost two thirds (64%) occurred in women (*4*). Similarly, a recent analysis of New York City's HIV reporting data found that 35% of new HIV diagnoses in 2001 were in women, compared with 28% before 2001 (*5*).

HIV infection in women has obvious implications for the health and well being of children. HIV infection can be transmitted perinatally, and increasing numbers of children—estimated at ≈12 million—are orphaned by the disease (*2*). Although preventing HIV transmission from an infected mother to her infant has become feasible due to effective antiretroviral treatment regimens and has met with great success in many parts of the world, services that prevent mother-to-child transmission are severely limited in low-income countries. Similarly, although combination antiretroviral therapy offers the potential to manage the disease as a chronic, treatable condition, access to such treatment is primarily limited to persons in high-income countries, which excludes the most severely affected regions. As an example, ≈4.1 million persons in Africa are in need of such therapy, but <2% have access to the drugs (*6*).

Preventing new infections is fundamental to stopping the spread of HIV. Attaining this goal requires that all persons have information about the disease and know their infection status, a formidable challenge in both low- and high-income countries. Such information can help uninfected persons remain free of the disease and help those who are infected gain access to treatment and prevent transmission to their partners. Fortunately, several broad-based national and international initiatives have been taken towards meeting these challenges. For example, the President's Emergency Plan for AIDS Relief is a 5-year, $15 billion commitment to treat HIV infection and prevent new infections in Africa and the Caribbean. Other undertakings include the United Nations' Global Fund to Fight AIDS, Tuberculosis, and Malaria and "The 3 by 5 Initiative," a detailed, multicountry plan developed by the World Health Organization and the Joint United Nations Programme on HIV/AIDS to provide antiretroviral treatment to 3 million HIV-infected persons in developing countries by the end of 2005.

## Malaria in Women

Malaria is another infectious disease threat that disproportionately affects women; it causes serious illness in pregnant women and children <5 years of age. Every year, malaria kills 1.5 million to 2.7 million persons and adversely affects another 300,000 to 500,000, mostly in Africa (*7**,**8*). Pregnant women suffer decreased immunity to malaria, which more than doubles their chances of contracting and dying of the disease (*9*). Pregnant women who contract malaria have an increased risk for severe maternal anemia. The consequent impaired fetal growth contributes to low birth weight in newborns. Malaria during pregnancy causes as many as 10,000 maternal deaths each year, 8%–14% of all low birthweight babies, and 3%–8% of all infant deaths in certain parts of Africa (*9*).

## Other Infectious Diseases in Women

In addition to HIV, women are more susceptible to other sexually transmitted diseases (STDs) and their long-term complications. In the United States >50% of preventable infertility is related to STDs (*10*). In addition, most sexually transmitted pathogens can be passed to the fetus or infant, sometimes with fatal consequences.

Longitudinal studies show that women are also at greater risk for active disease from *Mycobacterium tuberculosis* infection (*11*). Case-fatality rates are likewise higher in women. Reasons include decreased immune function due to poor nutritional status and delays in seeking care, both of which can be a function of gender.

The tropical parasitic disease schistosomiasis presents special concerns for women. Among parasitic diseases, schistosomiasis is second to malaria in prevalence; it affects >200 million persons in 74 countries (*12*). In affected areas, women are at greater risk for the disease compared to men because of their increased exposure to contaminated water through domestic work such as washing clothes and preparing food. Consequences of the disease are more severe in women than in men. Female genital schistosomiasis, often misdiagnosed as an STD, can cause tumors, ulcers, and infertility and may actually increase the risk for STDs (*13*).

## Infectious Diseases in Pregnant Women

Pregnancy complicates the impact of many other infectious diseases. Each year in the United States, ≈20,000 infants are born to women infected with hepatitis B virus (HBV) (*14*). Without postexposure prophylaxis, ≈6,000 of these infants would become chronically infected with HBV, and ≈1,500 would die prematurely of chronic liver disease. To address this problem, perinatal HBV prevention programs screen pregnant women for HBV and follow up with vaccination of newborns. Hepatitis C virus can also be transmitted during pregnancy, although the rate of infection appears lower than that of HBV. Hepatitis E virus can also have severe consequences if acquired during pregnancy, especially during the third trimester (*15**,**16*). It has been associated with increased risk for spontaneous abortions and still births as well as fulminant hepatitis in both mothers and infants (*16*).

Another serious maternal infection is group B streptococcus (GBS). GBS can be transmitted from mother to baby during pregnancy or during labor and delivery. During the 1990s, prevention efforts involving intrapartum antibiotic prophylaxis dramatically lowered the incidence of disease (*17*). However, GBS remains a leading infectious cause of illness and death among newborns in the United States (*18**,**19*).

## Reducing Health Disparities in Women

Women are caretakers and brokers of health for their families. These roles can increase their risk for infectious diseases and increase obstacles to adequate and timely treatment. Seeking health care can be the first step to identifying and treating a host of illnesses affecting women and their families. Therefore, innovative ways to reach at-risk women, including developing new research agendas to identify and address gender differences in infectious disease, are especially needed.

Reducing health disparities for women requires a multidisciplinary global effort to combat the root causes of these disparities—social, economic, and educational inequities that fuel the spread of diseases and perpetuate poverty throughout the world. Although much remains to be done, commitment to reduce these disparities on behalf of the international community is increasing. In addition, participants at the International Conference on Women and Infectious Diseases also play an important role in these efforts through their broad range of expertise and commitment to improving global health.
